# Evidence for Sprouting of Dopamine and Serotonin Axons in the Pallidum of Parkinsonian Monkeys

**DOI:** 10.3389/fnana.2018.00038

**Published:** 2018-05-15

**Authors:** Dave Gagnon, Lara Eid, Dymka Coudé, Carl Whissel, Thérèse Di Paolo, André Parent, Martin Parent

**Affiliations:** ^1^Department of Psychiatry and Neuroscience, Faculty of Medicine, CERVO Brain Research Centre, Université Laval, Quebec City, QC, Canada; ^2^Faculty of Pharmacy, Centre de Recherche du CHU de Québec, Université Laval, Quebec City, QC, Canada

**Keywords:** basal ganglia, globus pallidus, Parkinson's disease, MPTP-intoxicated monkeys, non-human primates, neurodegenerative diseases

## Abstract

This light and electron microscopie immunohistochemical quantitative study aimed at determining the state of the dopamine (DA) and serotonin (5-HT) innervations of the internal (GPi) and external (GPe) segments of the pallidum in cynomolgus monkeys (*Macaca fascicularis*) rendered parkinsonian by systemic injections of 1-methyl-4-phenyl-1,2,3,6-tetrahydropyridine (MPTP). In contrast to the prominent DA denervation of striatum, the GPi in MPTP monkeys was found to be markedly enriched in DA (TH+) axon varicosities. The posterior sensorimotor region of this major output structure of the basal ganglia was about 8 times more intensely innervated in MPTP monkeys (0.71 ± 0.08 × 10^6^ TH+ axon varicosities/mm^3^) than in controls (0.09 ± 0.01 × 10^6^). MPTP intoxication also induced a two-fold increase in the density of 5-HT (SERT+) axon varicosities in both GPe and GPi. This augmentation was particularly pronounced anteriorly in the so-called associative and limbic pallidal territories. The total length of the labeled pallidal axons was also significantly increased in MPTP monkeys compared to controls, but the number of DA and 5-HT axon varicosities per axon length unit remained the same in the two groups, indicating that the DA and 5-HT pallidal hyperinnervations seen in MPTP monkeys result from axon sprouting rather than from the appearance of newly formed axon varicosities on non-growing axons. At the ultrastructural level, pallidal TH+ and SERT+ axons were morphologically similar in MPTP and controls, and their synaptic incidence was very low suggesting a volumic mode of transmission. Altogether, our data reveal a significant sprouting of DA and 5-HT pallidal afferents in parkinsonian monkeys, the functional significance of which remains to be determined. We suggest that the marked DA hyperinnervation of the GPi represents a neuroadaptive change designed to normalize pallidal firing patterns associated with the delayed appearance of motor symptoms, whereas the 5-HT hyperinnervation might be involved in the early expression of non-motor symptoms in Parkinson's disease.

## Introduction

The main neuropathological hallmark of Parkinson's disease (PD) is a progressive degeneration of dopamine (DA) neurons located in the substantia nigra pars compacta (SNc) leading to a massive loss of DA input to the striatum. The decrease of striatal DA content is believed to be central in the expression of bradykinesia, resting tremor and rigidity that characterize PD. On the other hand, there is a growing interest in the fate of 5-hydroxytryptamine (serotonin or 5-HT) neurons in PD, mainly because of evidence that 5-HT striatal afferents are the main presynaptic determinant in the expression of L-3,4-dihydroxy-phenylalanine (L-Dopa)-induced dyskinesia (Carta et al., 2007), a motor disability characterized by abnormal involuntary movements that affect more than 75% of PD patients after only 15 years of dopatherapy (Yahr, 1972; Obeso et al., 2000; Rajput et al., 2002; Hely et al., 2005).

In addition to the striatum, the primate pallidum also receives significant DA and 5-HT inputs (Eid et al., 2013; Eid and Parent, 2015). However, by comparison to our knowledge of striatal DA and 5-HT involvement in the pathogenesis of PD, the possible contribution of such extrastriatal DA and 5-HT innervations is still unknown. Therefore, in the hope to shed a new light on the role of pallidal DA and 5-HT innervations in PD pathogenesis, we designed a study to determine the state of DA and 5-HT axonal projections to the internal (GPi) and external (GPe) segments of the pallidum in cynomolgus monkeys rendered parkinsonian after MPTP intoxication.

## Materials and methods

### Animals and behavioral assessment

Eight female cynomolgus monkeys (*Macaca fascicularis*, Primus Bio-Ressources inc.) of 4 years old, weighing between 2.8 and 3.9 kg were used. All monkeys were naïve and did not receive any other compounds unrelated to our study. For ethical and financial reasons, these monkeys were used in a previously published post-mortem analysis of the 5-HT striatal innervation (Gagnon et al., 2016), allowing direct comparisons with the present work dealing with pallidal innervation. Ovariectomy was performed on these animals to model the hormonal status of menopause women. This is particularly relevant to the fact that most parkinsonian women are menopause and that estrogens are known to be neuroprotective (Bourque et al., 2015). Animals were housed under a 12 h light-dark cycle with water and food *ad libitum*. All experimental procedures were approved by the *Comité de Protection des Animaux de l'Université Laval*, in accordance with the Canadian Council on Animal Care's Guide to the Care and Use of Experimental Animals (Ed2). Maximum efforts were made to minimize the number of animals used. Four monkeys received MPTP (Sigma-Aldrich Canada Ltd., Oakville, Canada) via a subcutaneous osmotic mini-pump for 14 days. Behavioural response to MPTP intoxication was assessed by using a faithful motor scale that takes into account posture, mobility, climbing, gait, grooming, voicing, social interaction and tremor (see Hadj Tahar et al., 2004 for details). Individual doses of MPTP as well as motor disability scores obtained for each monkey are given in Table 1.

**Table 1:** Specific information on control and MPTP-intoxicated monkeys.

**Group**	**Animal ID**	**Sex**	**Weight (kg)**	**Total MPTP (mg)**	Motor disability score^*^ (/16)	**Decrease of TH+ SNc neurons (%)**	**Decrease of TH striatal immunoreactivity^σ^ (%)**	**Decrease of DAT striatal immunoreactivity^σ^ (%)**
Control	S-2310	Female	3.9	NA	NA	NA	NA	NA
	S-2311	Female	3.0	NA	NA	NA	NA	NA
	S-2312	Female	3.5	NA	NA	NA	NA	NA
	S-2313	Female	3.8	NA	NA	NA	NA	NA
MPTP	S-2268	Female	3.2	6.00	8.23	82.5	87.9	87.2
	S-2269	Female	3.1	14.25	9.64	79.5	89.4	90.0
	S-2270	Female	2.8	11.50	6.78	70.5	84.0	83.4
	S-2273	Female	3.3	8.75	7.20	75.1	85.2	82.4

* *Behavioral response to MPTP intoxication was assessed by using the motor scale detailed in Hadj Tahar et al. (2004)*.

σ *Values are from the sensorimotor striatal territory (see Gagnon et al., 2016 for more details)*.

### Tissue preparation and immunohistochemistry

Five months after MPTP administration, animals were perfused transcardially as described in Eid and Parent (2017). Brains were then rapidly dissected out, post-fixed by immersion in 4% paraformaldehyde (PFA) for 24 h at 4°C and cut with a vibratome (model VT1200 S; Leica, Germany) into 50 μm-thick transverse sections collected in sodium phosphate-buffered saline (PBS).

The method used to assess the DA lesion induced by MPTP in these monkeys as been described elsewhere (Gagnon et al., 2016). Briefly, using standard immunoperoxidase method, equidistant transverse sections selected throughout the SNc and the striatum were immunostained for tyrosine hydroxylase (TH), the catalytic enzyme of DA synthesis, and/or the DA transporter (DAT), using 3,3′-diaminobenzidine tetrahydrochloride (DAB; catalog no. D5637; Sigma-Aldrich) as the chromagen. Sections taken through the SNc were immunostained for TH and counterstained with cresyl violet whereas striatal sections were stained for DAT and TH (see Table S1 for antibodies). Unbiased stereological counting of TH+ neurons was performed on SNc sections whereas striatal DA denervation was assessed using an infrared imaging system (Odyssey CLx; LI-COR Biosciences, Lincoln, NE, USA) and optical density measurements taken from delineated functional territories of the striatum (see Gagnon et al., 2016 for details).

The calbindin (CB) expression by TH immunoreactive neurons was assessed using a confocal imaging system (Zeiss, LSM 700; Oberkochen, Germany) and one double immunostained transverse section per brain taken through the SNc at −9 mm relative to the anterior commissure (Bowden and Martin, 2000) (see Table S1 for antibodies).

The number of tryptophan hydroxylase (TpH)-immunostained neurons of the dorsal raphe nucleus was assessed in each monkey using the immunoperoxidase methodological approach and unbiased stereology with a primary antibody against TpH, the rate-limiting enzyme in 5-HT synthesis, as described previously (Gagnon et al., 2016).

To assess the state of the DA innervation of the pallidum, a quantitative stereological analysis at the light microscope level was performed on 4 transverse sections taken through the pallidum (from −6 to 0 mm, relative to the anterior commissure, with a fixed interval of 1,800 μm) and immunostained for TH. In order to rule out the noradrenergic nature of TH+ axons observed in the pallidum, sections taken through the posterior GPi were doubly stained for TH and dopamine beta-hydroxylase (DBH), the enzyme that catalyze the hydroxylation of DA into noradrenaline, in both experimental groups. The absence of doubly-labeled neuronal element in the pallidum was confirmed using confocal microscopy (see Figure S1).

For electron microscopy, sections of the eight monkeys taken at −3 mm relative to the anterior commissure were also immunostained for TH, but in the absence of Triton X-100, which was replaced by 0.5% gelatin in all solutions. Sections were osmicated, dehydrated in ethanol and propylene oxide, and flat-embedded in Durcupan (catalog no. 44611-14; Fluka, Buchs, Switzerland) to be processed and examined as described below.

To provide a detailed and stereological quantitative description of the 5-HT axon distribution through the pallidum at the light microscope level, 10 transverse sections were selected across the entire pallidum (from −6 to 0 mm, relative to the anterior commissure), with a fixed interval of 600 μm. These sections were immunostained according to the immunoperoxidase method with an antibody against the 5-HT transporter (SERT, see Table S1). Other sections were also taken through the pallidum of all monkeys, at −3 mm relative to the anterior commissure, immunostained for SERT, but prepared for electron microscopy as described above.

### Stereology

Immunostained sections intended for stereology were examined under a light microscope equipped with a digital camera, a motorized stage and a Z-axis indicator controlled by a computer running StereoInvestigator software (v. 7.00.3; MicroBrightField, Colchester, VT, USA). The precise regional distribution of immunoreactive axons and axon terminals (varicosities) throughout the GPe and GPi was determined by dividing each pallidal segment into eight sectors, according to the method described in Eid et al. (2013). Numbers of TH-positive (+) and SERT+ axon varicosities were estimated using the optical fractionator probe (West et al., 1991) available in the StereoInvestigator software. Briefly, at each intersection of a grid formed by squares of 200 × 200 μm (TH+) and 600 × 600 μm (SERT+), a counting frame measuring 35 × 35 μm was drawn and examined with a 100x oil-immersion objective (NA 1.3, HCX PL Fluotar). Axon varicosities that fell inside the counting frame and did not contact the exclusion lines were counted whenever they came into focus within the 10 μm-thick disector. An average of 210 ± 17 (TH+) and 391 ± 23 (SERT+) varicosities were counted in each sector, yielding coefficients of error (Gundersen, *m* = 1 and 2nd Schmitz-Hof) between 0.03 and 0.22. Densities of TH+ and SERT+ varicosities were obtained using the total number of varicosities estimated by the optical disector and the volume of each sector estimated by Cavalieri's method.

The density of TH+ and SERT+ axons was assessed using the *spaceball* stereological probe (Mouton et al., 2002) that generates hemispheres on which a marker is placed when an immunolabeled axon intersects. For this experiment, 10 μm diameter hemispheres were randomly placed in the middle of the section at intersections of 200 × 200 μm grid for TH and 600 × 600 μm grid for SERT. The 100x oil-immersion objective was used for sampling. An average of 770 ± 39 (TH+) and 226 ± 13 (SERT+) markers were placed in each sector, yielding coefficients of error (Gundersen, m = 1 and 2nd Schmitz-Hof) between 0.05 and 0.46. Densities of TH+ and SERT+ axons were obtained using the total length of axons estimated by the spaceball probe and the volume of each sector estimated by Cavalieri's method. Thick TH+ or SERT+ axons could easily be distinguished from thin and varicose axons by their smooth aspect and their diameter larger than 1 μm.

### Ultrastructural analysis of TH+ and SERT+ axon varicosities

Quadrangular pieces were cut in the GPe and GPi of each monkey from flat-embedded TH or SERT-immunostained sections. After being glued on the tip of a resin block, they were cut at 80 nm with an ultramicrotome (model EM UC7, Leica). Ultrathin sections were collected on bare 150-mesh copper grids, stained with lead citrate and examined with a transmission electron microscope (Tecnai 12; Philips Electronic, 100 kV) and an integrated digital camera (XR-41, Advanced Microscopy Techniques). Axon varicosities were randomly sampled at a working magnification of 11,500x by taking a picture every time such profile was encountered until 45 or more pictures were available for analysis in each pallidal segment, for each monkey. Data from our previous experiments indicate that close examination of 45 profiles of a given type of axon varicosities is sufficient to provide a detailed description of its ultrastructural features (Eid et al., 2013, 2016; Eid and Parent, 2015; Gagnon et al., 2016). From these same photomicrographs, unlabeled axon varicosities were randomly selected for comparison. Morphometric features of labeled and unlabeled axon varicosities were analyzed using the ImageJ software (v 1.50b, NIH, USA) running a custom-made java application (Dave Gagnon: https://github.com/Fishwithatie/TEM_AnalysisWorkFlow). The synaptic incidence obtained from single-ultrathin sections was then extrapolated to the whole volume of varicosities by means of the formula of Beaudet and Sotelo (1981), using the long axis as diameter, according to Umbriaco et al. (1994).

### Statistical analysis

Because the data gathered from our 2 experimental groups, each composed of 4 animals, were not normally distributed, all statistical differences were assessed using a non-parametric statistical test, namely the Mann-Whitney *U*-test. Non-linear regressions were performed to analyse correlations between the density of axon varicosities and PD motor disability scores. Pearson correlation coefficient (*R*) was used to determine the correlation strength. Differences were considered statistically significant at *P* < 0.05. Statistical analysis was done using GraphPad Prism software (v. 6.01; GraphPad Software, San Diego, CA, USA). Mean and standard error of the mean are used throughout the text as central tendency and dispersion measure, respectively.

## Results

### MPTP intoxication induces a significant DA lesion and motor impairments

**Figure 1:**
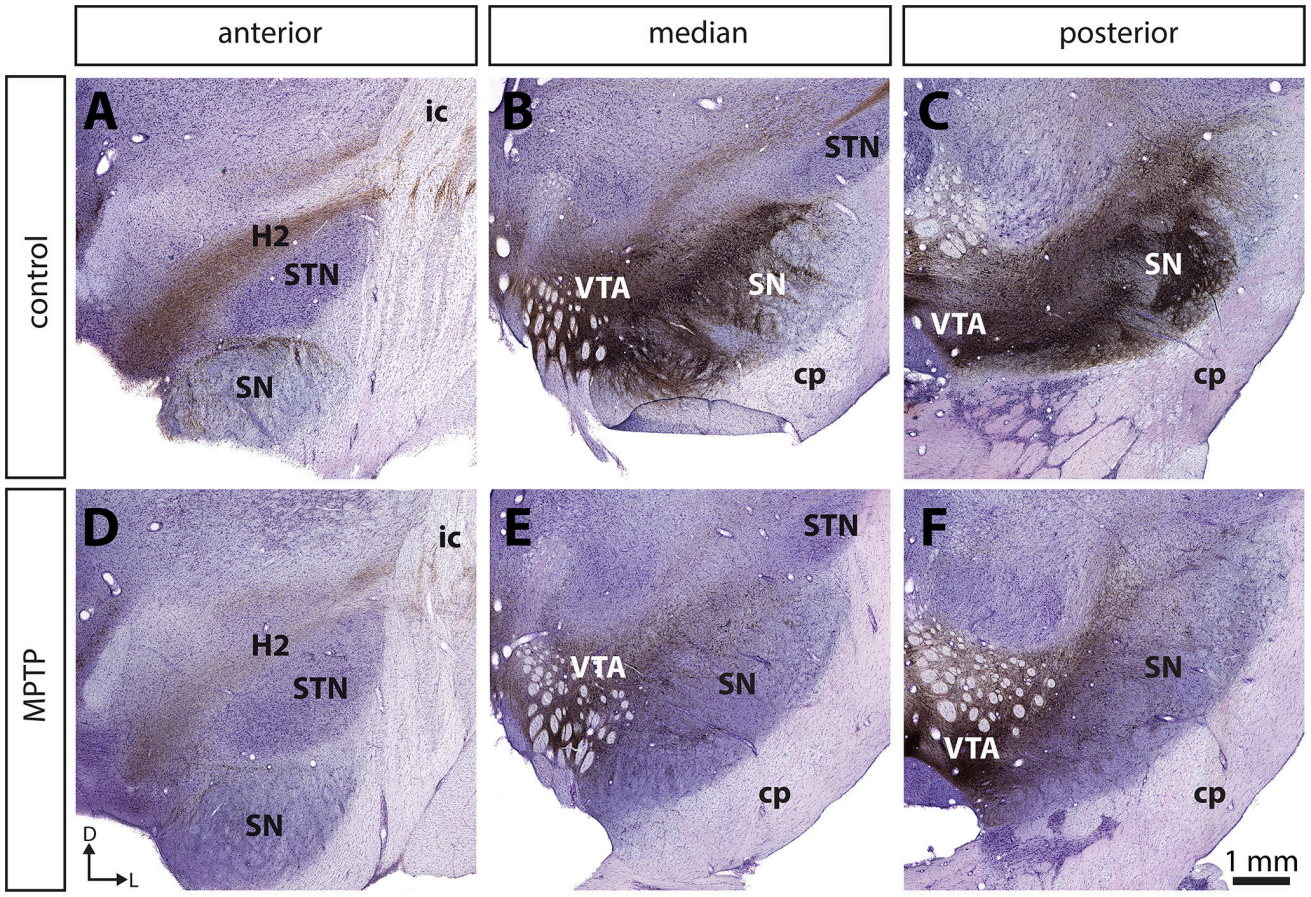
The number of tyrosine hydroxylase (TH) immunoreactive (+) neurons in the substantia nigra pars compacta (SNc) is significantly decreased in MPTP-intoxicated monkeys **(D–F)** compared to controls **(A–C)**. Transverse sections through the whole anteroposterior axis of the SNc and stained for tyrosine hydroxylase (TH, brown) and cresyl violet (purple) in control **(A–C)** and MPTP monkeys **(D–F)**. Our stereological analysis reveals a 77 % decrease in the number of TH+ neurons in the SNc of MPTP-intoxicated monkeys, compared to controls.

**Figure 2:**
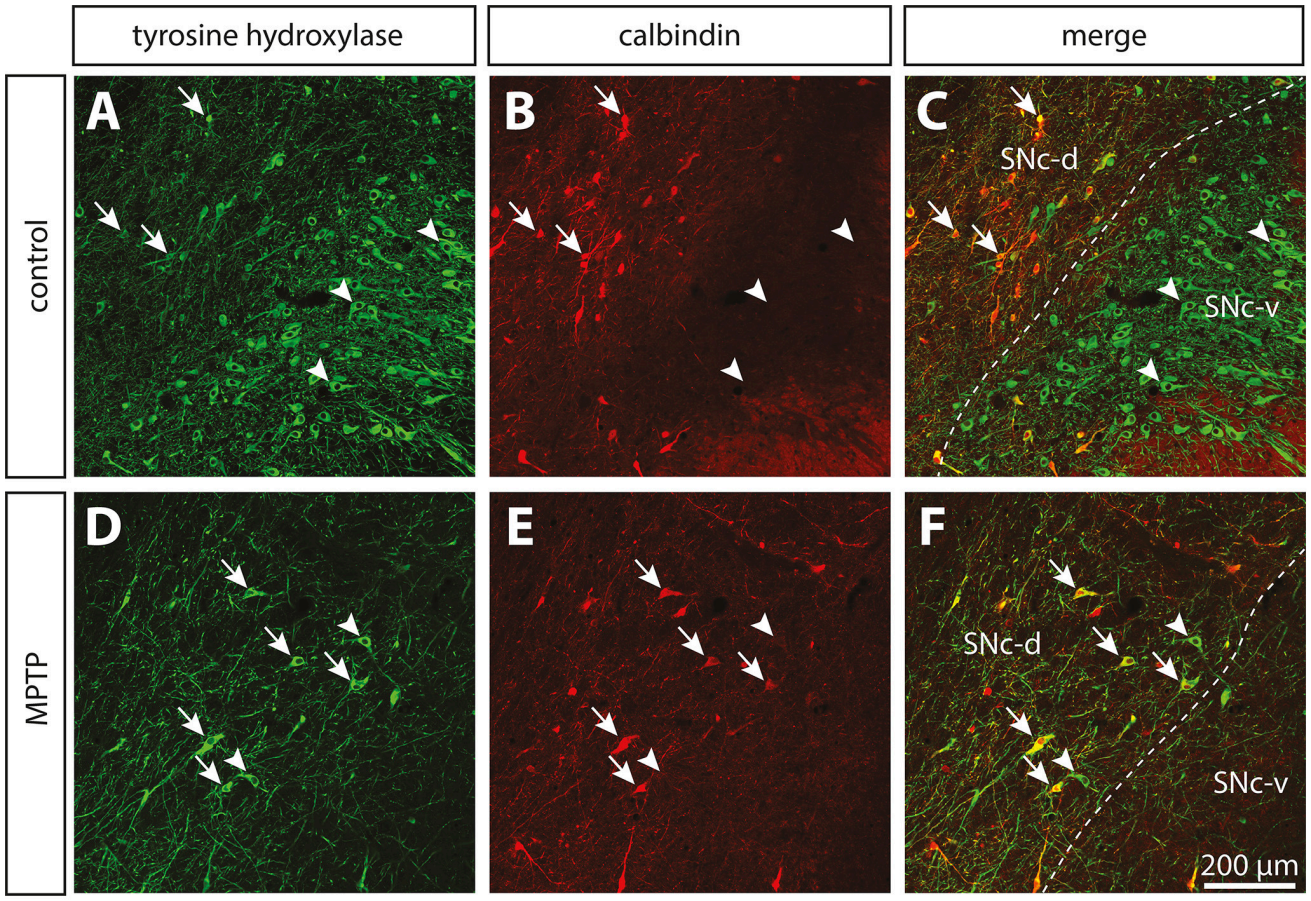
The dopamine neurons that are spared in the substantia nigra pars compacta (SNc) of MPTP-intoxicated monkeys are mainly located in the dorsal tier (SNc-d) and display calbindin (CB) immunoreactivity. Confocal images of transverse section through the SNc and immunostained for tyrosine hydroxylase (TH, green, **A,D**) and CB (red, **B,E**) in control **(A–C)** and MPTP monkeys **(D–F)**. Arrowheads point to neurons that express only TH, whereas arrows indicate SNc neurons that are immunoreactive for TH and CB.

Compared to controls, the number of TH+ neurons in the SNc of MPTP monkeys is decreased by 70.5–82.5% (Figure 1; see also Gagnon et al., 2016), leading to a 84.0–89.4% decline of TH and to a 82.4–90.0% diminution of DAT immunoreactivity in the sensorimotor functional territory of the striatum (Table 1). No significant changes were observed in the limbic striatal territory. Virtually all of the few TH+ neurons that remain in the SNc of MPTP monkeys are CB+ and they occur predominantly in the dorsal tier of the structure (Figure 2). There is no significant variation between controls and MPTP monkeys in regard to number of tryptophane hydroxylase-positive (TpH+) neurons in the dorsal raphe nucleus, with 5,355 ± 742 TpH+ neurons/mm^3^ of tissue in controls compared to 4,833 ± 354 in MPTP monkeys. Motor response to MPTP intoxication indicates scores ranging between 6.78/16 and 9.64/16, corresponding to moderate to severe PD states (see Table 1 for individual values).

### TH+ innervation of the pallidum in normal condition

**Figure 3:**
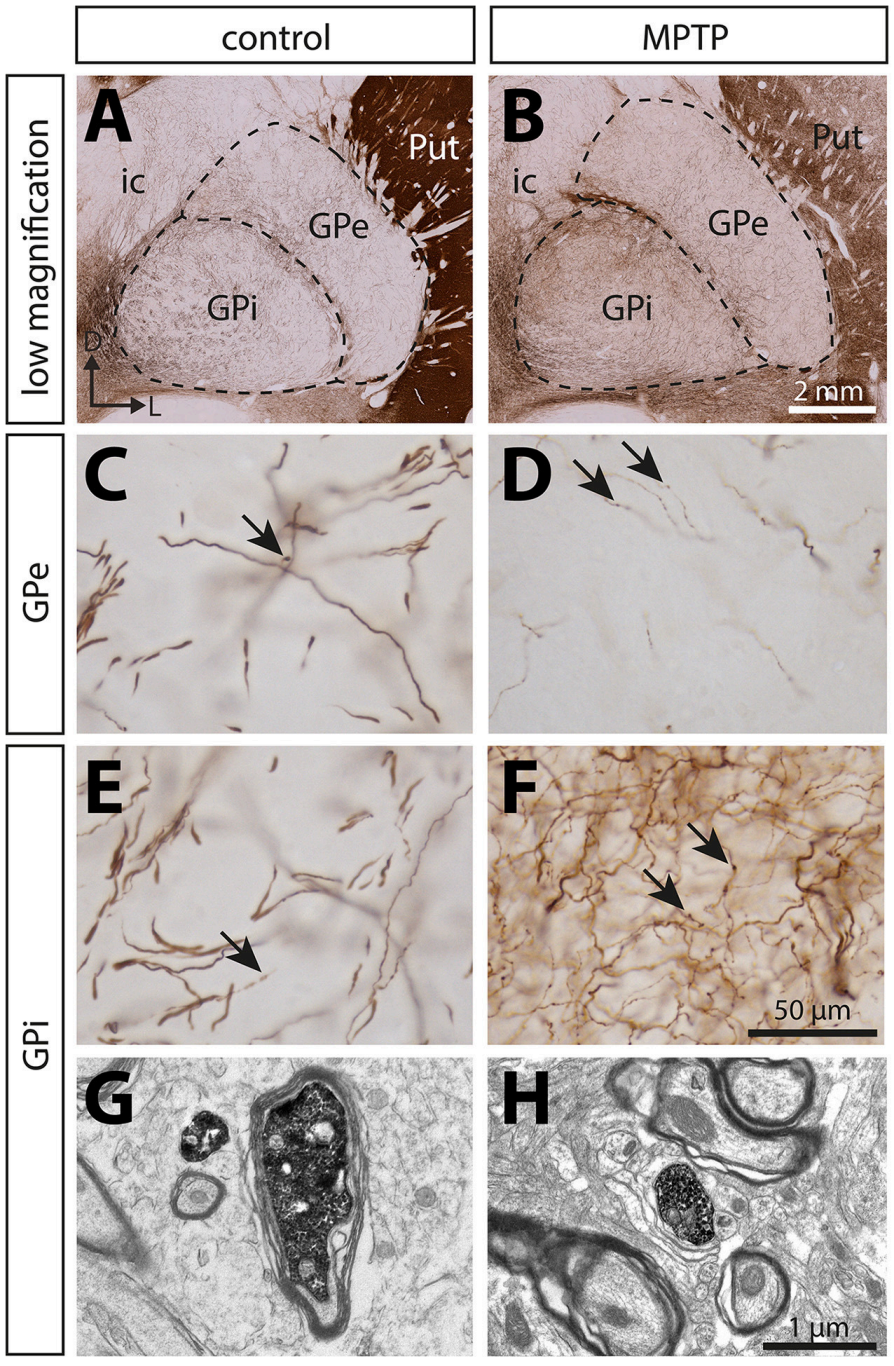
In contrast to the significant dopamine denervation of the putamen (Put), the immunoreactivity for tyrosine hydroxylase (TH) is significantly increased in the GPi of MPTP monkeys. Transverse sections through the GPe and GPi and immunostained for TH in controls **(A,C,E,G)** and MPTP monkeys **(B,D,F,H)**. Higher magnifications of TH+ axons observed in the GPe **(C,D)** and the GPi **(E,F)** are also provided. Arrows indicate examples of TH+ axon varicosities. Electron micrographs of TH+ axon profiles are also shown **(G,H)**. In contrast to MPTP-intoxicated monkeys, large and myelinated TH+ axons are often observed in control animals **(G)**. They likely correspond to the thick and non-varicose axons observed at the light microscope level, en route to the striatum.

At the light microscope level, the TH innervation of the pallidum appears to be composed of two types of TH+ axons: (a) small (0.52 ± 0.04 μm in diameter), highly varicose and tortuous, and (b) thick (1.63 ± 0.06 μm in diameter) and smooth (Figure 3). The thin and varicose axons represent 32% of all TH+ axons in the GPe and 37% in the GPi. The remaining thick and smooth axons travel mainly in bundles through a rather straight direction across both pallidal segments, en route to the putamen.

**Figure 4:**
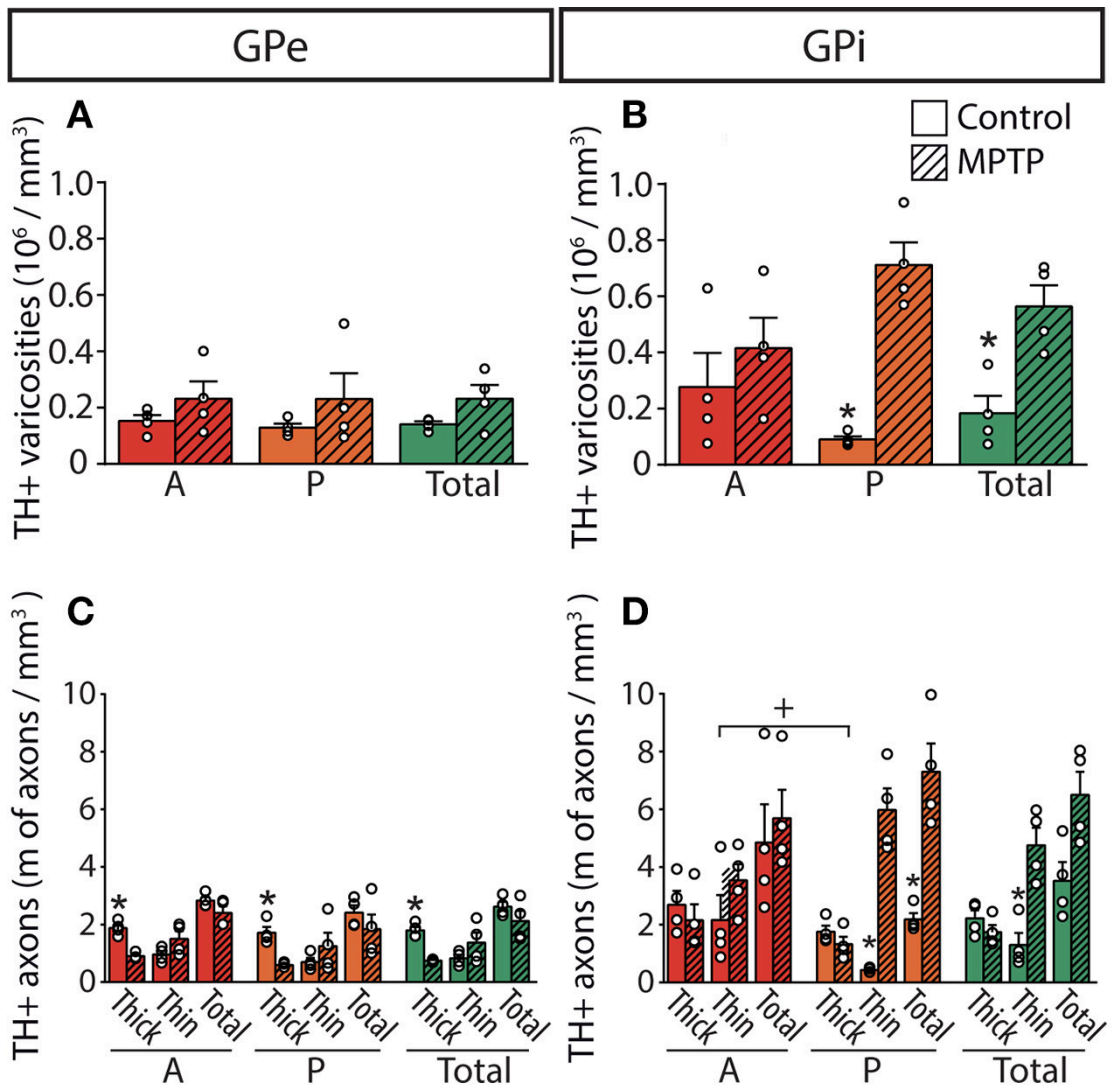
In MPTP monkeys, the axonal length of the thin and varicose TH+ axons increases significantly in the GPi whereas the length of the thick and non-varicose axons decreases in the GPe. Histograms showing the density of axon varicosities **(A,B)** as well as the axonal length **(C,D)** of the thin and the thick TH+ axons observed in the GPe **(A,C)** and GPi **(B,D)**. Dots represent individual values obtained for each monkey. ^*^P < 0.05 for MPTP vs. control monkeys, ^+^*P* < 0.05 for anterior (A) vs. posterior (P) pallidal sectors.

The DA innervation is similar in the two pallidal segments, with 0.14 ± 0.01 × 10^6^ TH+ varicosities/mm^3^ in GPe and 0.18 ± 0.06 × 10^6^ in GPi (Figure 4). A noticeable mediolateral-decreasing gradient of DA innervation is present in the GPe, with 0.19 ± 0.02 × 10^6^ TH+ axon varicosities/mm^3^ in the medial region compared to 0.09 ± 0.01 × 10^6^ in the lateral region (*P* = 0.0286). In contrast, the TH+ varicosities appear homogeneously distributed in the GPi. Estimates of the number of TH+ varicosities per 10 μm of axon were obtained by dividing the number of TH+ axon varicosities by the total length of the small caliber TH+ axons, and these values are similar in the GPe (1.90 ± 0.28 varicosities/10 μm of TH+ axons) and the GPi (1.79 ± 0.34, *P* = 0.8286).

### TH+ innervation of the pallidum in parkinsonian monkeys

The estimated volumes of the GPe and the GPi do not differ significantly between MPTP monkeys (53.24 ± 2.67 mm^3^ for GPe and 33.60 ± 2.80 mm^3^ for GPi) and controls (49.59 ± 3.76 mm^3^ for GPe and 37.15 ± 2.41 mm^3^ for GPi). In contrast to the striatum, which is markedly depleted of TH immunoreactivity in MPTP monkeys, the DA innervation of the pallidum is increased in all of its sectors. Such augmentation in the density of TH+ axon varicosities is particularly obvious in the posterior region of the GPi, with 0.71 ± 0.08 × 10^6^ TH+ axon varicosities/mm^3^ of tissue in MPTP monkeys compared to only 0.09 ± 0.01 × 10^6^ in controls (*P* = 0.0286, Figure 4).

The higher density of TH+ axon varicosities observed in the GPi after MPTP lesion is also reflected by the increase of the total length of the thin and varicose TH+ axons (4.8 ± 0.6 m of thin TH+ axons/mm^3^ of tissue in MPTP monkeys compared to only 1.3 ± 0.4 in controls, *P* = 0.0286). Again, this difference is more pronounced in the posterior region of the GPi with 6.0 ± 0.8 m of thin TH+ axons/mm^3^ in MPTP monkeys compared to only 0.43 ± 0.04 in controls (*P* = 0.0286). In accordance with a higher density of TH+ axon varicosities and TH+ thin axons, the number of varicosities/10 μm of small-caliber TH+ axons does not change significantly after MPTP administration in both pallidal segments, suggesting that the increase of the density of TH+ axon varicosities is caused by the sprouting of varicose TH+ axons rather than by the appearance of newly formed axon varicosities on non-growing axons. In contrast to the density of the thin and varicose axons that increases significantly in the GPi of MPTP monkeys, the density of the thick and smooth TH+ axons appears similar. Interestingly, the two MPTP-intoxicated monkeys with the lowest motor disability scores (6.78 and 7.20) show the largest increase in density of TH+ axons and axon varicosities in the GPi. This observation suggests a possible negative correlation between motor disability score and DA GPi hyperinnervation that would become statistically significant with a larger sample (*R* = −0.8660, *P* = 0.1922, Figure S2).

In contrast to the GPi, the density of the thick and smooth TH+ axons in the GPe, which are probably heading to the striatum, is significantly reduced after MPTP intoxication reaching 0.75 ± 0.02 m of TH+ axons/mm^3^ of tissue in MPTP monkeys compared to 1.8 ± 0.1 in controls (*P* = 0.0286), whereas the density of the thin and varicose TH+ axons appears unaltered following MPTP administration. The TH+ axons present in the pallidum do not display DBH immunoreactivity, a finding that dismisses their possible noradrenergic nature (Figure S1).

### Ultrastructural features of TH+ axon varicosities

When examined at the ultrastructural level, the two types of TH+ fibers detected in the GPi of normal monkeys (thin and varicose versus large and smooth) stand out as small unmyelinated axons and large myelinated axons, respectively (Figures 3G,H). In the GPi of MPTP monkeys, only thin unmyelinated TH+ axons are observed. No significant difference regarding the size and shape of TH+ axon varicosities can be found between MPTP and control monkeys. When extrapolated to the whole volume of varicosities by means of the stereological formula of Beaudet and Sotelo (1981), similar synaptic incidences are noted for TH+ axon varicosities observed in the two experimental groups (7 ± 3% in the GPi of MPTP monkeys compared to 13 ± 7% in control animals, when extrapolated to the whole volume of axon varicosities). In comparison, the synaptic incidence of unlabeled, randomly selected axon varicosities in the GPi of control animals was 28 ± 3% (Table 2). All synaptic contacts made by TH+ axon varicosities occur on dendritic profiles.

**Table 2:** Morphometric characteristics of TH+ axon varicosities in the GPi of control and MPTP monkeys.

	**GPi**			
	**Control (*n* = 4)**		**MPTP (*n* = 4)**	
	**TH**	**Unlabeled**	**TH**	**Unlabeled**
Numbered examined	269	267	285	286
**Dimensions**				
Short axis (μm)	0.60 ± 0.04^*^	0.43 ± 0.01	0.47 ± 0.03	0.45 ± 0.02
Long axis (μm)	1.23 ± 0.11^*^^+^	0.73 ± 0.03	0.89 ± 0.07	0.75 ± 0.03
Diameter (μm)	0.92 ± 0.08^*^^+^	0.58 ± 0.02	0.68 ± 0.05	0.60 ± 0.03
Aspect ratio	0.56 ± 0.02+	0.62 ± 0.01	0.58 ± 0.01^*^	0.62 ± 0.01
Area (μm^2^)	0.72 ± 0.11^*^^+^	0.27 ± 0.02	0.37 ± 0.05	0.29 ± 0.02
% with mitochondria	65 ± 4^*^	40 ± 5	77 ± 18	45 ± 5
**Synaptic incidence (%)**				
Single section	3 ± 1^*^	10 ± 1	2 ± 1	4 ± 2
Whole volume	13 ± 7	28 ± 3	7 ± 3	8 ± 4
Length of synaptic juction (μm)	0.19 ± 0.06	0.26 ± 0.02	0.31 ± 0.11	0.34 ± 0.14
**Junctions**				
Symmetrical	0 ± 0^+^	35 ± 22	67 ± 33	57 ± 30
Asymmetrical	100 ± 0^+^	65 ± 22	33 ± 33	43 ± 30

*Data are presented as means ± SEM. The unlabeled profiles were selected at random from the same micrographs displaying TH+ profiles*.

* *P = 0.0248 for TH+ vs. unlabeled*,

+ *P = 0.0248 for SERT+ vs. TH+*.

### 5-HT innervation of the pallidum in normal condition

**Figure 5:**
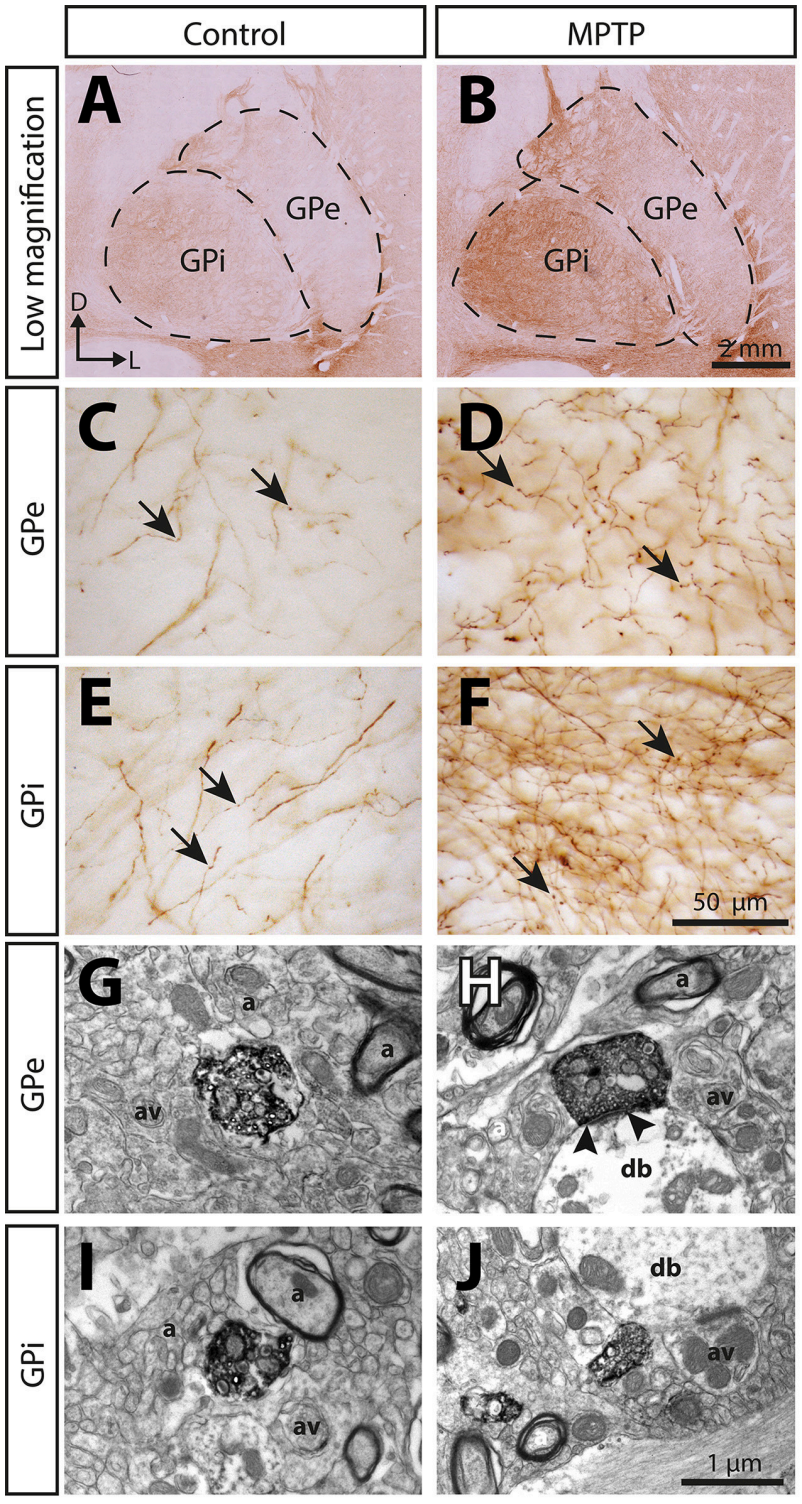
The immunoreactivity for the serotonin membrane transporter (SERT) is significantly increased in the pallidum of MPTP-intoxicated monkeys. Transverse sections through the GPe and the GPi and immunostained for SERT in control **(A,C,E,G,I)** and MPTP monkeys **(B,D,F,H,J)**. Higher magnifications of SERT+ axons observed in the GPe **(C,D)** and the GPi **(E,F)** are also provided. Arrows indicate examples of SERT+ axon varicosities. Electron micrographs of SERT+ axon varicosity profiles are also shown **(G–J)**. SERT+ axon varicosities are often observed apposed to small myelinated or unmyelinated axons (a) and axon varicosities (av). The SERT+ axon varicosity profile depicted in H establishes an asymmetric synaptic contact (between arrow heads) with a pallidal dendritic branch (db) whereas the three others **(G,I,J)** are devoid of synaptic membrane specialization.

The 5-HT innervation of the pallidum is mainly composed of thin (0.5 ± 0.1 μm) and varicose SERT+ axons that display a tortuous course without any preferential orientation (Figure 5). Using a stereological approach, we estimate that, in control animals, this type of axons represents 92% of SERT+ axons in the GPe and 93% in the GPi. The remaining axons are thick (1.1 ± 0.1 μm), do not show varicosity and do not travel in bundle or show any preferential orientation.

**Figure 6:**
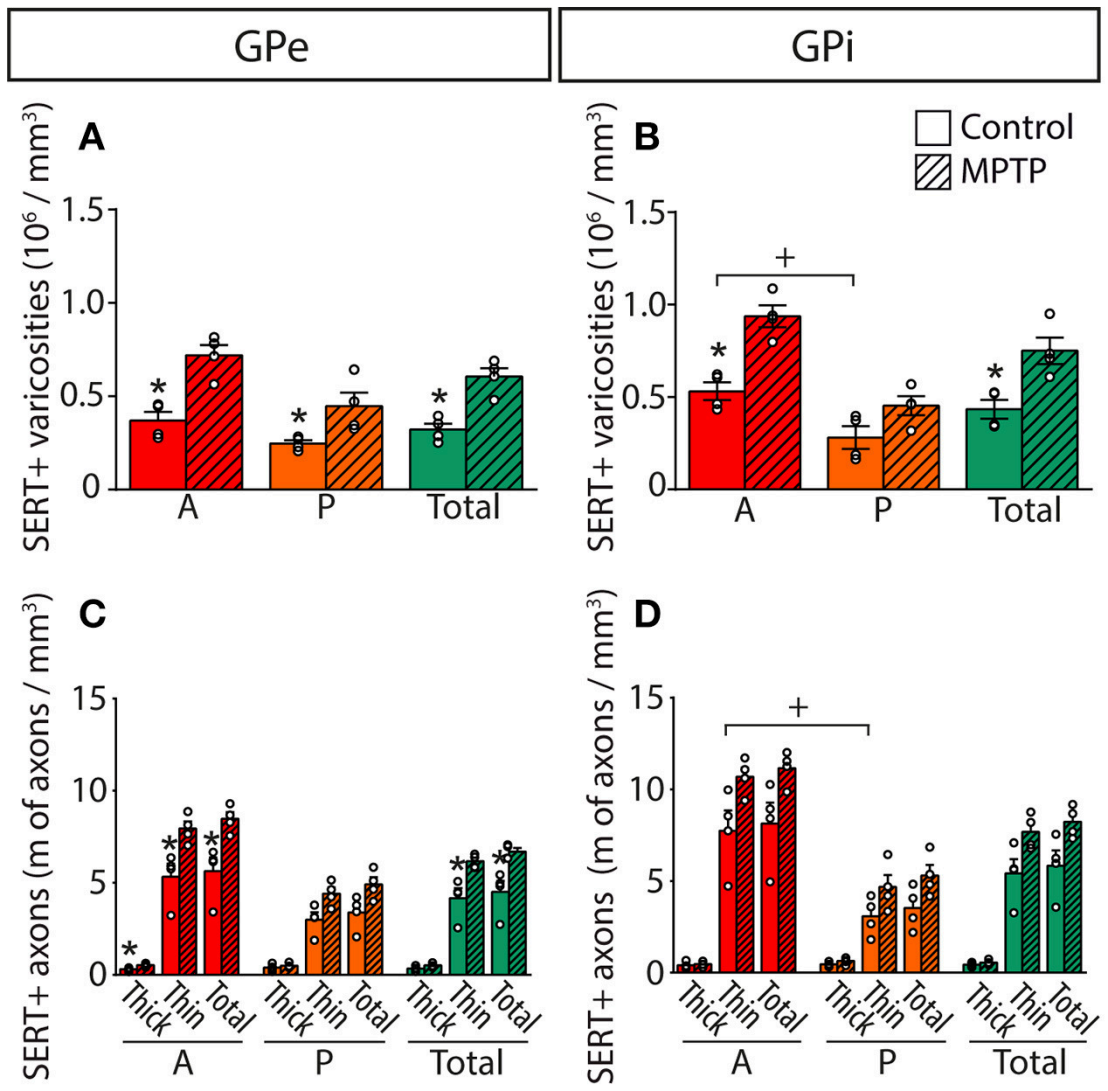
In MPTP monkeys, the densities of SERT+ axon varicosities and SERT+ axons are significantly increased in the GPe and GPi, particularly in their anterior sectors. Histograms showing the density of SERT+ axon varicosities **(A,B)** as well as the axonal length **(C,D)** of the thick and thin SERT+ axons observed in the GPe **(A,C)** and the GPi **(B,D)**. Dots represent individual values obtained for each monkey. ^*^P < 0.05 for MPTP vs. control monkeys, ^+^*P* < 0.05 for anterior vs. posterior pallidal sectors.

Overall, the density of SERT+ axon varicosities appears similar between the GPe (0.32 ± 0.05 × 10^6^ SERT+ axon varicosities/mm^3^ of tissue) and the GPi (0.43 ± 0.05 × 10^6^, Figure 6). Both pallidal segments display an anteroposterior-decreasing gradient of 5-HT innervation that becomes significant only in the GPi with 0.53 ± 0.05 × 10^6^ SERT+ axon varicosities/mm^3^ of tissue in its anterior part compared to 0.28 ± 0.06 × 10^6^ in its posterior region (*P* = 0.0286). This gradient is also reflected by unbiased estimations of the density of SERT+ axons indicating that, in contrast to the thick SERT+ axons that are homogeneously distributed through both pallidal segments, the thin and varicose SERT+ axons are more abundant in the anterior part of the GPi (7.7 ± 1.1 m of axons/mm^3^ of tissue) than in posterior region (3.1 ± 0.5, *P* = 0.0286). By dividing the estimated number of SERT+ axon varicosities by the total length of small caliber SERT+ axons, we can approximate the number of SERT+ varicosities per 10 μm of axon. These values are similar in the GPe (0.84 ± 0.08 SERT+ axon varicosities/10 μm of axon) and the GPi (0.84 ± 0.11), as well as across different pallidal regions. It is noteworthy that SERT+ pallidal afferents display a lower number of varicosities/10 μm of axon than the TH+ pallidal projections.

In the GPe of control animals, the density of SERT+ axon varicosity is significantly higher than that of TH+ (0.32 ± 0.03 × 10^6^ SERT+ axon varicosities/mm^3^ vs. 0.14 ± 0.01 × 10^6^ TH+ axon varicosities/mm^3^, *P* = 0.0286). This difference becomes particularly significant in the anterior part of the GPe, which harbors 0.37 ± 0.05 × 10^6^ SERT+ varicosities/mm^3^ compared to 0.15 ± 0.02 × 10^6^ TH+ varicosities/mm^3^ (*P* = 0.0286). In the GPi, only the posterior region shows higher density for SERT+ axon varicosities with 0.28 ± 0.06 × 10^6^/mm^3^ vs. 0.09 ± 0.01 × 10^6^ axon varicosities/mm^3^ for TH+ axon varicosities (*P* = 0.0286).

### 5-HT innervation of the pallidum in parkinsonian monkeys

In MPTP-intoxicated monkeys, the density of SERT+ axon varicosities is increased by 91% in the GPe to reach 0.61 ± 0.05 × 10^6^ SERT+ varicosities/mm^3^ (*P* = 0.0286) and by 74% the GPi to reach 0.75 ± 0.07 × 10^6^ SERT+ varicosities/mm^3^ (*P* = 0.0286). This augmentation is more significant in the anterior sectors of the GPe with 0.72 ± 0.06 × 10^6^ SERT+ varicosities/mm^3^, representing a two-fold increase compared to control animals (0.37 ± 0.05 × 10^6^). In the GPi, the increased density of SERT+ axon varicosities is statistically significant only in anterior sectors with 0.94 ± 0.06 × 10^6^ SERT+ varicosities/mm^3^ compared to 0.53 ± 0.05 × 10^6^ in control animals (*P* = 0.0286).

The higher density of SERT+ axon varicosities in the pallidum of MPTP monkeys is also reflected by a significant increase of the density of the thin and varicose SERT+ axons in the GPe (6.2 ± 0.2 m of SERT+ axons/mm^3^ of tissue in MPTP monkeys compared to 4.2 ± 0.6 in controls, *P* = 0.0286). Such a gain does not reach statistical significance in the GPi (7.7 ± 0.5 in MPTP animals compared to 5.4 ± 0.8 in controls). In the GPe, increase in the density of the thin and varicose SERT+ axons is significant in the anterior sectors (7.9 ± 0.4 m of SERT+ axons/mm^3^ of tissue in MPTP monkeys compared to 5.3 ± 0.7 in controls, *P* = 0.0286) and in the medial sectors (7.6 ± 0.3 m of SERT+ axons/mm^3^ of tissue in MPTP monkeys compared to 4.7 ± 0.7 in control animals, *P* = 0.0286, Figure 6). In accordance with a higher density of SERT+ axon varicosities and SERT+ thin axons, no significant differences between MPTP and control monkeys are observed when considering the number of SERT+ varicosities/10 μm of axon in the GPe nor the GPi. Also, there is no significant change in regard to the density of the thick SERT+ axons in the GPe and the GPi, and no statistically significant correlation could be made between the increased SERT+ pallidal innervation reported above for the density of axon varicosities in MPTP monkeys and their PD motor disability scores.

### Ultrastructural features of SERT+ axon varicosities

At the electron microscope level, SERT+ axon varicosities derive exclusively from unmyelinated axons and the ones present in the GPe and GPi of MPTP monkeys are similar in size and shape to those in controls (Figures 5G–J, Table 3). These axon varicosities are usually ovoid, contain aggregated and small vesicles and often display one or more mitochondria. Statistical analysis of morphometric measurements, such as diameter and cross-sectional area, reveals that SERT+ axon varicosities in both GPe and GPi are similar in MPTP and control animals, and are also similar to randomly selected, unlabeled axon varicosity profiles.

**Table 3:** Morphometric characteristics of SERT+ axon varicosities in the GPe and GPi of control and MPTP monkeys.

	**GPe**				**GPi**			
	**Control (*n* = 4)**		**MPTP (*n* = 4)**		**Control (*n* = 4)**		**MPTP (*n* = 4)**	
	**SERT**	**Unlabeled**	**SERT**	**Unlabeled**	**SERT**	**Unlabeled**	**SERT**	**Unlabeled**
Number examined	213	185	212	195	216	198	238	214
**Dimensions**								
Short axis (μm)	0.55 ± 0.05	0.49 ± 0.01	0.48 ± 0.03	0.50 ± 0.03	0.49 ± 0.03	0.50 ± 0.03	0.46 ± 0.03	0.48 ± 0.01
Long axis (μm)	0.94 ± 0.13	0.75 ± 0.04	0.77 ± 0.06	0.75 ± 0.04	0.79 ± 0.05	0.75 ± 0.02	0.77 ± 0.03	0.76 ± 0.02
Diameter (μm)	0.74 ± 0.09	0.62 ± 0.02	0.62 ± 0.04	0.63 ± 0.03	0.64 ± 0.04	0.62 ± 0.02	0.61 ± 0.03	0.62 ± 0.01
Aspect ratio	1.75 ± 0.11	1.58 ± 0.05	1.64 ± 0.03	1.54 ± 0.05	1.64 ± 0.03	1.58 ± 0.05	1.72 ± 0.04	1.62 ± 0.02
Area (μm2)	0.51 ± 0.09	0.35 ± 0.03	0.38 ± 0.06	0.36 ± 0.03	0.39 ± 0.05	0.36 ± 0.02	0.35 ± 0.04	0.35 ± 0.03
% with mitochondria	72 ± 3^*^	59 ± 4	62 ± 5	52 ± 2	79 ± 3^*^	55 ± 3	68 ± 5	60 ± 4
**Synaptic incidence (%)**								
Single section	7 ± 2^*^	24 ± 5	7 ± 2^*^	20 ± 1	7 ± 1^*^	23 ± 1	5 ± 1^*^	17 ± 3
Whole volume	31 ± 10	80 ± 22	21 ± 7^*^	69 ± 9	23 ± 2^*^	77 ± 6	16 ± 3^*^	64 ± 9
Length of synaptic junction (μm)	0.26 ± 0.05	0.25 ± 0.02	0.29 ± 0.06	0.22 ± 0.02	0.25 ± 0.03	0.22 ± 0.01	0.27 ± 0.03	0.20 ± 0.01
**Junctions**								
Symmetrical	48 ± 5^*^	83 ± 3	35 ± 22	87 ± 3	63 ± 14	96 ± 4	60 ± 16	92 ± 5
Asymmetrical	52 ± 5	17 ± 3	65 ± 22	13 ± 3	38 ± 14	4 ± 4	40 ± 16	8 ± 5

*Data are presented as means ± SEM. The unlabeled profiles were selected at random from the same micrographs displaying SERT+ profiles*.

* *P = 0.0248 for SERT+ vs. unlabeled*.

Interestingly, in both pallidal segments of control and MPTP monkeys, very few SERT+ axon varicosities display a synaptic contact when compared to unlabeled profiles (*P* = 0.0248). The synaptic incidence of the SERT+ axon varicosities is estimated at 31 ± 10% in the GPe and 23 ± 2% in GPi compared to 80 ± 22 and 77 ± 6% for unlabeled axon varicosities (Table 3). The synaptic incidence of the SERT+ axon varicosity profiles is similar to the one reported above for the TH+ profiles and, like TH+ axon varicosities, significantly lower than unlabeled counterparts. These values indicate that approximately 70% of SERT+ axon varicosities present in both pallidal segments are devoid of any synaptic specialization. Interestingly, there is no significant difference between MPTP and control monkeys regarding the synaptic incidence of SERT+ axon varicosities present in the GPe and GPi (21 ± 7 vs. 31 ± 10% in the GPe and 16 ± 3% vs. 23 ± 2% in the GPi). In both pallidal segments of control and MPTP monkeys, synapses formed by SERT+ axon varicosities occur exclusively on pallidal dendrites and are of the symmetrical or asymmetrical types.

## Discussion

This study provides the first detailed quantitative and ultrastructural analysis of neuroadaptive changes displayed by the DA and 5-HT pallidal afferents in a non-human primate model of PD. Our stereological estimations show a significant DA hyperinnervation of the GPi in MPTP monkeys that contrasts strikingly with the massive degeneration of the nigrostriatal pathway. We also report a significant increase of the 5-HT pallidal innervation in MPTP monkeys compared to controls, particularly in the anterior pallidal sectors, where the associative and limbic functional territories reside. The DA hyperinnervation is overall more pronounced than that of 5-HT and occurs mainly within the posterior regions of the GPi, where the sensorimotor territory is located. We argue that these significant neuroadaptive changes of the DA and 5-HT pallidal innervations play a significant role in delayed expression of motor symptoms and early expression of non-motor symptoms of PD, respectively.

### The morphological characteristics of dopamine and serotonin pallidal afferents

The density of the DA pallidal innervation in cynomolgus monkeys was found to be similar in the GPe and GPi. Both pallidal segments harbor numerous thin and varicose axons together with a smaller number of thick and smooth axons, as is also the case in the globus pallidus of rats (Rodrigo et al., 1998; Fuchs and Hauber, 2004; Debeir et al., 2005), squirrel monkeys (Eid and Parent, 2015), African green monkeys (Jan et al., 2000) and human (Prensa et al., 2000). When examined under the electron microscope, the thick and smooth axons were found to be heavily myelinated, whereas the thin and varicose axons were devoid of myelin sheath. The course and morphological aspect of these two types of fibers indicate that the thick and smooth ones are fibers of passage en route to the striatum, whereas the thin and varicose axons are the ones that arborize locally within the pallidum. The synaptic incidence displayed by the thin and varicose fibers was found to be very low in comparison to that of unlabeled axon varicosities present in the same microenvironment.

The density of axons and axon varicosities of the 5-HT type is also similar in the two pallidal segments. Although the 5-HT pallidal input in primates is known to be overall more prominent than the DA input (Eid et al., 2013; Eid and Parent, 2015, 2016), a detailed comparison of the GPe and GPi of the cynomolgus monkey reveal that the two types of innervations display a similar proportion in each pallidal segment. Our electron microscopie findings indicate that, as it is the case for the DA input, the 5-HT influence upon pallidal neurons is largely mediated through an asynaptic mode of transmission.

### The DA pallidal innervation is significantly increased in parkinsonian monkeys

The present study provides the first quantitative demonstration that the MPTP lesion of the DA nigrostriatal pathway induces a significant DA hyperinnervation in the GPi, particularly in its posterior regions where the sensorimotor territory is located.

Previous works on this issue have led to conflicting results. Some studies indicate that the DA pallidal innervation in MPTP monkeys is preserved (Parent et al., 1990; Schneider and Dacko, 1991; Parent and Lavoie, 1993; Mounayar et al., 2007; Dopeso-Reyes et al., 2014; Ballanger et al., 2016), whereas others suggest that this type of innervation is decreased (Pifl et al., 1991, 1992; Jan et al., 2000). In human, positron emitting tomography (PET) study of the brain of PD patients indicate that the DA pallidal innervation is either preserved (Lewis et al., 2012) or increased (Whone et al., 2003; Moore et al., 2008; Pavese et al., 2012). The latter findings are at variance with the biochemical decrease in DA concentrations detected in post-mortem samples of the pallidum of PD patients (Hornykiewicz, 1966; Bernheimer et al., 1973; Ploska et al., 1982; Jan et al., 2000; Rajput et al., 2008). However, the information yielded by such post-mortem investigations is limited to only one precise moment in the disease progression, often its end-stage, and does not provide clues as to how the various neuronal systems rearrange themselves during the earlier phases of the pathology. Interestingly, PET studies indicate that the increase of the DA pallidal innervation occurs in the early stage of disease (Whone et al., 2003; Moore et al., 2008; Pavese et al., 2011) and that the loss of DA pallidal innervation in latter periods may represent a pivotal step in disease progression. We therefore presume that the DA sprouting in the GPi detected in the present study occurs rapidly after MPTP intoxication. We see such a sprouting as a mechanism designed to compensate the MPTP-induced degeneration of the DA nigrostriatal pathway that could play a significant role in delaying the expression of motor symptoms. Under certain conditions, such a compensatory mechanism might lead to a partial recovery of motor functions, until the surviving DA neurons are no longer able to maintain a functional level of DA in the pallidum.

The low synaptic incidence of DA as well as 5-HT axon varicosities has been viewed as the morphological evidence of diffuse (volumic) transmission. The 8-fold increase of DA innervation that occurred in the sensorimotor region of the GPi in MPTP monkeys, as reported here, indicates that, in this particular pathological condition, the diffuse mode of transmission might indeed contribute to the maintenance of a functional DA ambient level in the pallidum. Such neuroadaptive changes of the nigropallidal projection can be seen as part of a compensatory mechanism designed to delay the onset of PD motor symptoms. Such a view is indeed supported by a trend toward a negative correlation (*R* = −0.87) between the severity of motor symptoms and the level of sprouting of pallidal DA axons observed in our 4 MPTP monkeys. Also congruent with this hypothesis is the fact that injections of glial-cell-line-derived neurotrophic factor (GDNF) in the caudate nucleus and the SNc of MPTP monkeys lead to significant increase in pallidal DA levels, along with a certain degree of motor recovery (Gash et al., 1996). Furthermore, intra-pallidal (GPe) injections of DA antagonists were shown to worsen PD symptoms in monkeys that have recovered from MPTP-intoxication (Neumane et al., 2012). Altogether, these data indicate that DA hyperinnervation of the pallidum may lead to a delayed onset of PD motor symptoms that would explain why motor deficits do not appear before a 70–90% decrease of striatal DA content occurs in PD patients (Bernheimer et al., 1973; Riederer and Wuketich, 1976) as well as in MPTP-intoxicated monkeys (German et al., 1988; Bezard et al., 2001; Meissner et al., 2003).

The robust increase of the GPi DA innervation is in striking contrast with the 80% loss of DA neurons that we noted in the SNc of MPTP monkeys. This finding indicates that the remaining DA midbrain neurons undergo an important morphological reorganization, including a significant sprouting of their axon projecting to the pallidum. These surviving neurons were found to abound principally in the dorsal tier of SNc and to express CB, in agreement with previous post-mortem studies undertaken in other MPTP monkeys (Parent et al., 1990; Lavoie and Parent, 1991; Parent and Lavoie, 1993; Dopeso-Reyes et al., 2014) or in PD human brains (Yamada et al., 1990; German et al., 1992; Hurley et al., 2013). Altogether, these findings have led to the hypothesis that CB could exert a neuroprotective effect upon the DA SNc neurons at the origin of the nigropallidal DA projection. Indeed, the fact that the severe reduction of the striatal DA innervation is paralleled by a marked hypertrophy of the DA pallidal innervation in MPTP monkeys favors the idea of a distinct origin and a lesser susceptibility to neurotoxic insults of the nigropallidal DA projection. In support of a specific origin of the nigropallidal projection are the results of a previous single-axon study of SNc and retrorubral field projection neurons in African green monkeys, which reveal that such axons innervate either the pallidum or the striatum, but not both structures (Jan et al., 2000). Beside the presence of CB, the difference in length and degree of axonal arborisation may also explain why the nigrostriatal pathway is more prone to degenerate than the nigropallidal projection. As we have argued elsewhere (Parent and Parent, 2006), the maintenance of the morphological and functional integrity of the nigrostriatal DA pathway, which comprises long, highly collateralized and widely distributed axons, is extremely demanding in terms of metabolic energy and this renders such axons highly vulnerable to the neurodegenerative processes at play in PD (see also Bolam and Pissadaki, 2012). Such an energetic burden would not be as great for the nigropallidal projection, which is composed of shorter, thinner and less profusely arborized axons than those of the nigrostriatal pathway. In line with such a view, the thick and myelinated DA pallidal axons, which are most likely nigrostriatal fibers en route to the striatum, were found to be specifically targeted in our MPTP monkeys, whereas the thin and varicose DA pallidal axons are those that proliferate in the same animals.

The functional impact of the increase of DA innervation of GPi in PD condition is currently unknown, but this neuroadaptive change could represent a compensatory mechanism designed to lower the GPi neuronal activity that is abnormally augmented in PD animal models (see above). Indeed, DA could exert a presynaptic inhibitory effect on GPi neurons through the activation of the D_1_ excitatory receptor located on striatal GABAergic inhibitory afferents to the pallidum, facilitating GABA release in the GPi (Kliem et al., 2007, 2010). The D_2_ inhibitory DA receptor, which is also expressed presynaptically in the GPi, could allow DA to inhibit GPi neurons by reducing the release of glutamate by pallidal afferents originating from the subthalamic nucleus (Hadipour-Niktarash et al., 2012).

### The 5-HT pallidal innervation is significantly increased in parkinsonian monkeys

The present stereological analysis reveals a 91% increase of the density of 5-HT axon varicosities in the GPe compared to a 74% augmentation in the GPi of MPTP monkeys. The fate of the 5-HT pallidal innervation in MPTP monkeys has been the subject of only a few studies, some reporting a preservation (Zeng et al., 2010) and others a decrease of this type of innervation (Rylander et al., 2010). Such a discrepancy might reflect variations in the animal survival time, which did not allow the documentation of compensatory mechanisms that are likely to be more prominent early in the evolution of PD symptoms (our monkeys were perfused 5 months after MPTP intoxication) than in later phases of the disease. Studies of the state of the striatal 5-HT innervation in animal models of PD have yielded much more consistent results, with a sprouting of the 5-HT striatal axons reported in 6-OHDA rats (Zhou et al., 1991; Guerra et al., 1997; Maeda et al., 2003, 2005), 6-OHDA mice (Bez et al., 2016), MPTP mice (Rozas et al., 1998), as well as MPTP monkeys (Gaspar et al., 1993; Zeng et al., 2010; Gagnon et al., 2016).

Stereological analyses of the dorsal raphe nucleus in our MPTP monkeys reveal that the density and morphological features of 5-HT cell bodies are not affected (Gagnon et al., 2016), a finding that supports the idea of a rearrangement of the surviving 5-HT neurons and the sprouting of their varicose axons in the pallidum. Such a sprouting of 5-HT axons, as observed in the GPe, the GPi (current study) and the striatum (Gagnon et al., 2016), suggests that these axons originate from 5-HT neurons endowed with a highly collateralized axon that undergo a significant morphological rearrangement in PD condition. Congruent with such a view, our previous single-axon tracing experiments in rats have shown that most neurons of the dorsal raphe nucleus that send their axon to the pallidum also project to the striatum through a widely distributed set of collaterals (Gagnon and Parent, 2014). Moreover, the fact that the 5-HT axon varicosities observed in the putamen and the pallidum share similar morphological features, including their size, also favors the hypothesis that they arise from the same neuronal population located in the dorsal raphe nucleus.

The 5-HT axons are known to be able to metabolize L-Dopa administered to PD patients into DA, thanks to their content in aromatic L-amino acid decarboxylase (Arai et al., 1994). They can also store and release DA via the vesicular monoamine transporter 2 (Ng et al., 1970, 1971; Hollister et al., 1979; Arai et al., 1994, 1995; Tanaka et al., 1999; Maeda et al., 2005). However, being devoid of the D_2_ autoreceptor and DAT, 5-HT axons release DA in a non-physiological manner. This phenomenon occurring in the DA-denervated striatum has been viewed as the main presynaptic determinant of L-Dopa-induced dyskinesia (reviewed in Carta et al., 2008; Carta and Tronci, 2014). The significant DA hyperinnervation of the GPi in MPTP monkeys, as reported here, led us to believe that the role played by 5-HT axons in the uncontrolled release of DA following L-Dopa administration at pallidal level is not as significant as what might occur in the striatum where a severe DA denervation is observed.

The fact that the highest increase of pallidal 5-HT innervation in MPTP monkeys occurs in the limbic and associative functional territories of this basal ganglia component reinforces the notion of the involvement of 5-HT in the expression of non-motor symptoms of PD, such as depression, apathy and anxiety that precede, sometimes by several years, the expression of motor symptoms (Schapira et al., 2017). We hypothesize that the increase of the 5-HT pallidal tone most likely influences other pallidal inputs, including the GABAergic afferent projections arising from the striatum that are known to express the presynaptic inhibitory receptor 5-HT_1B_ (Bonaventure et al., 1998; Castro et al., 1998; Riad et al., 2000; Sari, 2004; Mostany et al., 2005). This adaptation would therefore significantly contribute to the disinhibition of the GPi, whose neuronal activity is increased in PD (Miller and Delong, 1987; Filion and Tremblay, 1991; Wichmann et al., 1999). Congruent with such a view is the finding that 5-HT_1B_ receptor agonists increase the firing rate of both GPi and GPe neurons in monkeys (Kita et al., 2007).

Despite a significant augmentation of the pallidal 5-HT innervation, levels of 5-HT receptors, including 5-HT_1A_ (Riahi et al., 2012), 5-HT_1B_ (Riahi et al., 2013), and 5-HT_2A_ (Riahi et al., 2011), are not significantly altered in MPTP monkeys, suggesting that the effect on pallidal neural activity is mainly mediated through an increase in the local 5-HT concentration rather than a rise of the expression of 5-HT receptor by pallidal neurons or their afferent projections. The low synaptic incidence of the 5-HT innervation of the primate pallidum might be the ideal morphological substratum for such an elevation of 5-HT local pallidal concentration in MPTP monkeys. We estimate that approximately 70% of 5-HT axon varicosities observed in the GPe and the GPi are in fact devoid of any synaptic contact (see also Eid et al., 2013). This finding can be viewed as the morphological evidence of the existence of a diffuse mode of 5-HT transmission (reviewed in Descarries et al., 2010), allowing the maintenance of a local concentration of 5-HT susceptible to play a significant role in the functional organization of the pallidum under normal and pathological conditions.

## Conclusions

We report significant neuroadaptive changes of the DA and 5-HT pallidal afferent projections in PD monkeys. On the one hand, we hypothesize that the sprouting of DA axons in the sensorimotor territory of the GPi is an early compensatory mechanism designed to restore normal GPi activity and involved in the delayed appearance of motor symptoms. On the other hand, we suggest that the sprouting of 5-HT axons in the associative and limbic pallidal territories is involved in the appearance of non-motor symptoms of PD that precede, often by several years, the expression of motor symptoms.

## Author contributions

DG conducted most of the experiments, images acquisition, analyses and data interpretation. LE, DC, and CW contributed to experiments. TD provided expertise with behavioral characterization of MPTP monkeys. AP provided critical comments on the manuscript. MP is the principal investigator who designed the study and wrote the manuscript.

### Conflict of interest statement

The authors declare that the research was conducted in the absence of any commercial or financial relationships that could be construed as a potential conflict of interest.

## Abbreviations

5-HT: Serotonin
a: Axon
A: Anterior
av: Axon varicosity
CB: Calbindin
cp: Cerebral peduncle
db: Dendritic branch
DBH: Dopamine beta-hydroxylase
DA: Dopamine
DAB: 3,3′diaminobenzidine tetrahydrochloride
DAT: Dopamine transporter
GDNF: Glial-cell-line-derived neurotrophic factor
GPe: External segment of the globus pallidus
GPi: Internal segment of the globus pallidus
H2: Lenticular fasciculus
ic: Internal capsule
L-Dopa: L-3,4-dihydroxy-phenylalanine
MPTP: 1-methyl-4-phenyl-1,2,3,6-tetrahydropyridine
P: Posterior
PBS: Sodium phosphate-buffered saline
PD: Parkinson's disease
PET: Positron emission tomography
PFA: Paraformaldehyde
Put: Putamen
SERT: Serotonin transporter
SN: Substantia nigra
SNc: Substantia nigra pars compacta
STN: Subthalamic nucleus
TH: Tyrosine hydroxylase
TpH: Tryptophan hydroxylase
VTA: Ventral tegmental area.
