# Supplementary material for: Evidence for Sprouting of Dopamine and Serotonin Axons in the Pallidum of Parkinsonian Monkeys
Source: Front Neuroanat. 2018 May 15;12:38. doi: 10.3389/fnana.2018.00038 (PMC5963193; doi:10.3389/fnana.2018.00038)
Supplement: Supplementary file 1 [file Data_Sheet_1.pdf]

Supplementary Material

Evidence for sprouting of dopamine and serotonin axons in the pallidum of parkinsonian monkeys

Dave Gagnon, Lara Eid, Dymka Coudé, Carl Whissel, Thérèse Di Paolo, André Parent, Martin Parent*

*** Correspondence:** Martin Parent: martin.parent@fmed.ulaval.ca

1 Supplementary Figures and Table

1.1 Supplementary Figures

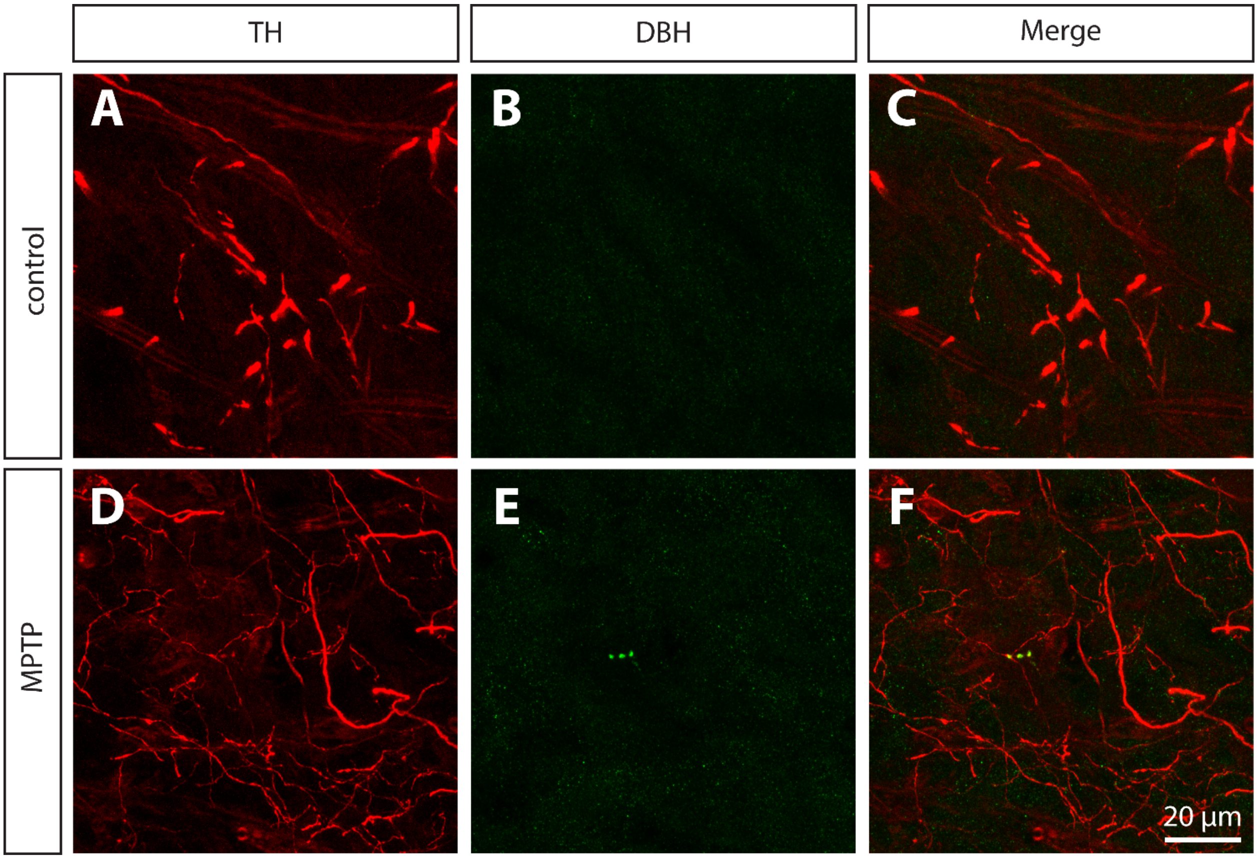

Supplementary Figure 1. Tyrosine hydroxylase (TH) immunoreactive axons observed in the pallidum of control and MPTP-intoxicated monkeys are devoid of immunoreactivity for dopamine beta-hydroxylase (DBH), ruling out their noradrenergic nature. Confocal images of transverse sections taken through the posterior GPi and immunostained for TH (red, A, D) and DBH (green, B, E) in control (A-C) and MPTP (D-F) monkeys.

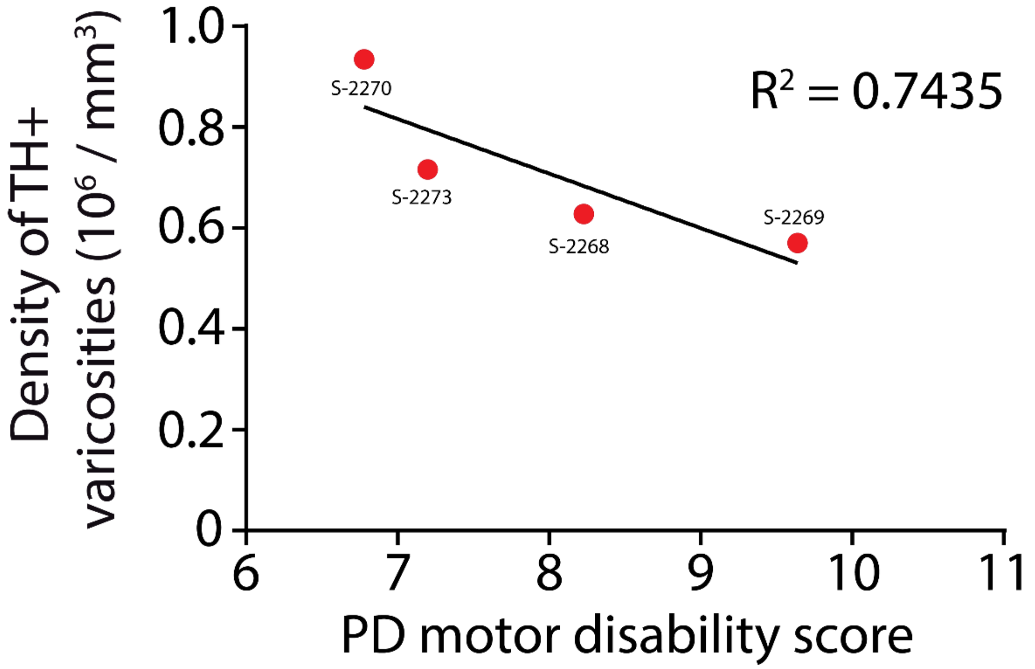

Supplementary Figure 2. Correlation between densities of tyrosine hydroxylase (TH) immunoreactive axon varicosities in the posterior GPi, as estimated using unbiased stereological approach in each monkey, and their motor disability scores, as assessed with the motor scale detailed in Hadj Tahar et al. 2004.

1.2 Supplementary Table

Table S1 Specific information on antibodies used

Staining	Primary antibody	Product #	Dilution	Secondary antibody	Product #	Dilution
TH staining of SNc neurons	Mouse α TH	**22941;** Immunostar, Hudson, USA	1 / 1000	Biotinylated Horse α mouse	**BA-2000;** Vector Laboratories, Burlingame, CA, USA	1 / 1000
TH and CB double staining of SNc neurons	Rabbit α TH	**AB151;** Chemicon International Inc., Temecula, USA	1 / 1000	Donkey α rabbit 488	**711-545-152;** Jackson ImmunoResearch Inc., West Grove, USA	1 / 1000
	Mouse α CB	**C-9848;** Sigma-Aldrich, Oakville, Canada	1 / 1000	Donkey α mouse Cy5	**715-175-150;** Jackson ImmunoResearch Inc., West Grove, USA	1 / 1000
TH and DAT double staining of striatal axons	Mouse α TH	**22941;** Immunostar, Hudson, USA	1 / 1000	Goat α mouse 680	**926-68070;** LI-COR Biosciences, Lincoln, NE, USA	1 / 1000
	Rat α DAT	**MAB369;** EMD Millipore Corporation, Billerica, USA	1 / 500	Goat α rat 800	**926-32219;** LI-COR Biosciences, Lincoln, NE, USA	1 / 1000
TpH staining of dorsal raphe neurons	Sheep α TpH	**AB1541;** EMD Millipore Corporation, Billerica, USA	1 / 250	Biotinylated rabbit α sheep	**BA-6000;** Vector Laboratories, Burlingame, CA, USA	1 / 200
SERT staining of GPe and GPi axons	Goat α SERT	**SC-1458;** Santa Cruz Biotechnology, Dallas, TX, USA	1 / 1000	Biotinylated rabbit α goat	**BA-5000;** Vector Laboratories, Burlingame, CA, USA	1 / 1000
TH staining of GPe and GPi axons	Mouse α TH	**22941;** Immunostar, Hudson, USA	1 / 1000	Biotinylated horse α mouse	**BA-2000;** Vector Laboratories, Burlingame, CA, USA	1 / 200
TH and DBH double staining of GPe and GPi axons	Mouse α TH	**22941;** Immunostar, Hudson, USA	1 / 1000	Donkey α mouse 594	**715-585-150;** Jackson ImmunoResearch Inc., West Grove, USA	1 / 200
	Rabbit α DBH	**AB1536;** Chemicon International Inc., Temecula, USA	1 / 250	Donkey α rabbit 488	**711-545-152;** Jackson ImmunoResearch Inc., West Grove, USA	1 / 200

All primary antibodies were incubated overnight at 4°C. All secondary antibodies were incubated at room temperature during 2h.
